# Factors predicting regression of visual acuity following successful treatment of anisometropic amblyopia

**DOI:** 10.3389/fmed.2022.1013136

**Published:** 2022-10-31

**Authors:** Yu Jia, Jing Liu, Qingqing Ye, Shenglan Zhang, Lei Feng, Zixuan Xu, Yijing Zhuang, Yunsi He, Yusong Zhou, Xiaolan Chen, Ying Yao, Rengang Jiang, Benjamin Thompson, Jinrong Li

**Affiliations:** ^1^State Key Laboratory of Ophthalmology, Zhongshan Ophthalmic Center, Sun Yat-Sen University, Guangzhou, China; ^2^Centre for Eye and Vision Research, Hong Kong, Hong Kong SAR, China; ^3^School of Optometry and Vision Science, University of Waterloo, Waterloo, ON, Canada; ^4^Liggins Institute, University of Auckland, Auckland, New Zealand

**Keywords:** anisometropic amblyopia, treated amblyopia, regression, risk factors, prediction model

## Abstract

**Objective:**

To identify factors associated with visual acuity regression following successful treatment of anisometropic amblyopia.

**Design and method:**

This was a retrospective cohort study. Database records for 100 and 61 children with anisometropic amblyopia who met at least one criterion for successful treatment proposed by the Pediatric Eye Disease Investigator Group (PEDIG) and had at least 1 year of follow-up data available after the criterion was met were analyzed. The study sample was split into two groups, those who regressed within 1 year of successful treatment (no longer met any of the PEDIG criteria for successful treatment) and those who did not. A two-step analysis involving a least absolute shrinkage and selection operator (LASSO) regression and a logistic regression were used to identify predictor variables for increased risk of regression. A broad range of clinical, perceptual, and demographic variables were included in the analyses.

**Results:**

Sixty-eight (42.5%) children regressed within 1 year of successful treatment. Among the 27 predictor variables considered within the statistical modeling process, the three most important for predicting treatment regression were the extent of amblyopic eye visual acuity improvement, age at first hospital visit and sex. Specifically, lower risk of regression was associated with larger amblyopic eye visual acuity improvement with treatment, younger age at initiation of treatment and female sex.

**Conclusion:**

Patients who received treatment at a younger age and responded well to treatment had a lower risk of treatment regression. This pattern of results suggests that early detection of amblyopia and strategies that enhance treatment adherence may reduce the risk of treatment regression. The higher risk of regression in boys than girls that we observed may reflect known sex differences in brain development and /or sex differences in environment within our sample of children from South China.

## Introduction

Amblyopia is defined clinically as reduced visual acuity in an otherwise healthy eye combined with a history of disrupted visual experience early in life (1). Typical causes of disruption include bilateral refractive error, anisometropia, strabismus, visual deprivation, or a combination of these conditions. In childhood and adolescence, the visual acuity deficit that characterizes amblyopia can be treated with refractive correction and, where indicated, occlusion or penalization of the stronger or non-amblyopic fellow eye for several hours each day (2–5). These treatments alter visual input to the brain and, *via* mechanisms that are not yet understood, improve neural processing of information from the amblyopic eye.

Although available treatments for the visual acuity deficit in amblyopia are effective, regression of visual acuity when treatment is discontinued is a concern. Estimates of visual acuity regression rates following treatment cessation vary substantially from <10% (6) of patients to between 20 to 40% (7–9) and even over 40% (10, 11) depending on the study population, definition of regression and duration of follow-up. Regression may or may not be influenced by tapering of treatment (8, 12) and is not related to binocular status at the end of treatment (13).

Several prospective and retrospective follow-up studies have explored factors that may predict visual acuity regression following amblyopia treatment, but a clear pattern of risk factors has yet to emerge. Risk factors that may be important include greater patient age at diagnosis (14), younger age at cessation of occlusion therapy (7), better visual acuity at the time of treatment cessation (13, 14), poorer visual acuity at the start of treatment (11, 15), larger visual acuity gains during treatment (13, 14), high levels of anisometropia (10, 15, 16) combined with microtropia (17) or strabismus (11, 18), and a prior history of treatment regression (13).

In this study, we took advantage of criteria for defining amblyopia treatment success used by the Pediatric Eye Disease and Investigator Group (PEDIG) and a unique database of amblyopia treatment records (19) to retrospectively investigate risk factors for amblyopia regression in patients with successfully treated anisometropic amblyopia. To determine amblyopia treatment success, we combined criteria used across different PEDIG amblyopia treatment studies. We considered amblyopia treatment to be successful if any of the following three criteria were met at 3–12 months follow-up: interocular difference (IOD) in acuity <0.2 logMAR (referred to as the IOD criterion) (4), best corrected visual acuity (BCVA) improvement of 3 or more logMAR lines (the BCVA improvement criterion) (20, 21) or, amblyopic eye visual acuity ≤ 0.1 logMAR (the BCVA achievement criterion) (20, 21). We applied the PEDIG amblyopia treatment success criteria to records of patients with anisometropic amblyopia within the Uniting Functions in Ophthalmology and Optometry (UFOs) Database (19) that is held at Zhongshan Ophthalmic Center, a major regional eye hospital in Guangzhou, China. The database contains longitudinal clinical and visual function data for every amblyopia patient seen at the hospital. Once we had identified patients with anisometropic amblyopia who met the PEDIG treatment success criteria, we were able to compare patients who regressed (no longer met the PEDIG criteria) with those who did not within a 1-year time window to identify risk factors for regression.

## Materials and methods

This study followed the tenets of the Declaration of Helsinki and was approved by the Institutional Ethics Committee of Zhongshan Ophthalmic Center, Sun Yat-Sen University.

### Participants

The UFOs Database was searched for records of patients with amblyopia treated between January 2019 and July 2021. Inclusion criteria for our study were: (1) a diagnosis of monocular amblyopia defined as an interocular difference in best corrected visual acuity ≥0.30 logMAR that could not be explained by pathology, (2) meeting at least one of the PEDIG criteria for amblyopia treatment success following amblyopia treatment, and (3) availability of follow-up data for at least 1 year after the PEDIG criterion was met. Participants in the database all received amblyopia treatment in accordance with AAO guidelines (1, 22). This involved the correction of refractive error followed by occlusion or penalization of the fellow eye if required. Patient records were excluded if they included a history of strabismus, visual deprivation, cataract, aphakia, pseudophakia or any other ocular or neurological disorder. These exclusion criteria meant that only patients with anisometropic amblyopia were included in the study sample. Anisometropia was defined as the interocular difference in refractive error ≥-2.0 D for myopia, ≥1.5 D for hyperopia and ≥2.0 D for astigmatism.

### Vision measures

All participants underwent regular ocular health and vision assessments at Zhongshan Ophthalmic Center (one visit every 3 months was recommended). The assessments followed standard operating procedures to reduce inter-tester variability. Measurements included a routine ocular examination, measurement of undilated spherical and cylindrical refractive error (KR-8800, Topcon Corp, Tokyo, Japan; dilated refractive error measurements were available from the first hospital visit), best corrected monocular distance visual acuity (logMAR tumbling E), contrast sensitivity [psychophysical quick contrast sensitivity function (qCSF) (23) for those older than 6 years, the CSV-1000E for those 6 and under] and stereoacuity (Random Dot Stereoacuity at near and the Randot Stereoacuity Test at distance). Spherocylinder values were calculated as the sum of the absolute sphere and cylinder values. Contrast sensitivity measures were converted to measures of area under the contrast sensitivity function (AUCSF) (24). Nil stereopsis was recorded as 5,000 arc sec (25). Full details of the standardized testing protocol have been reported previously (19).

### Statistical analysis

**Figure 1:**
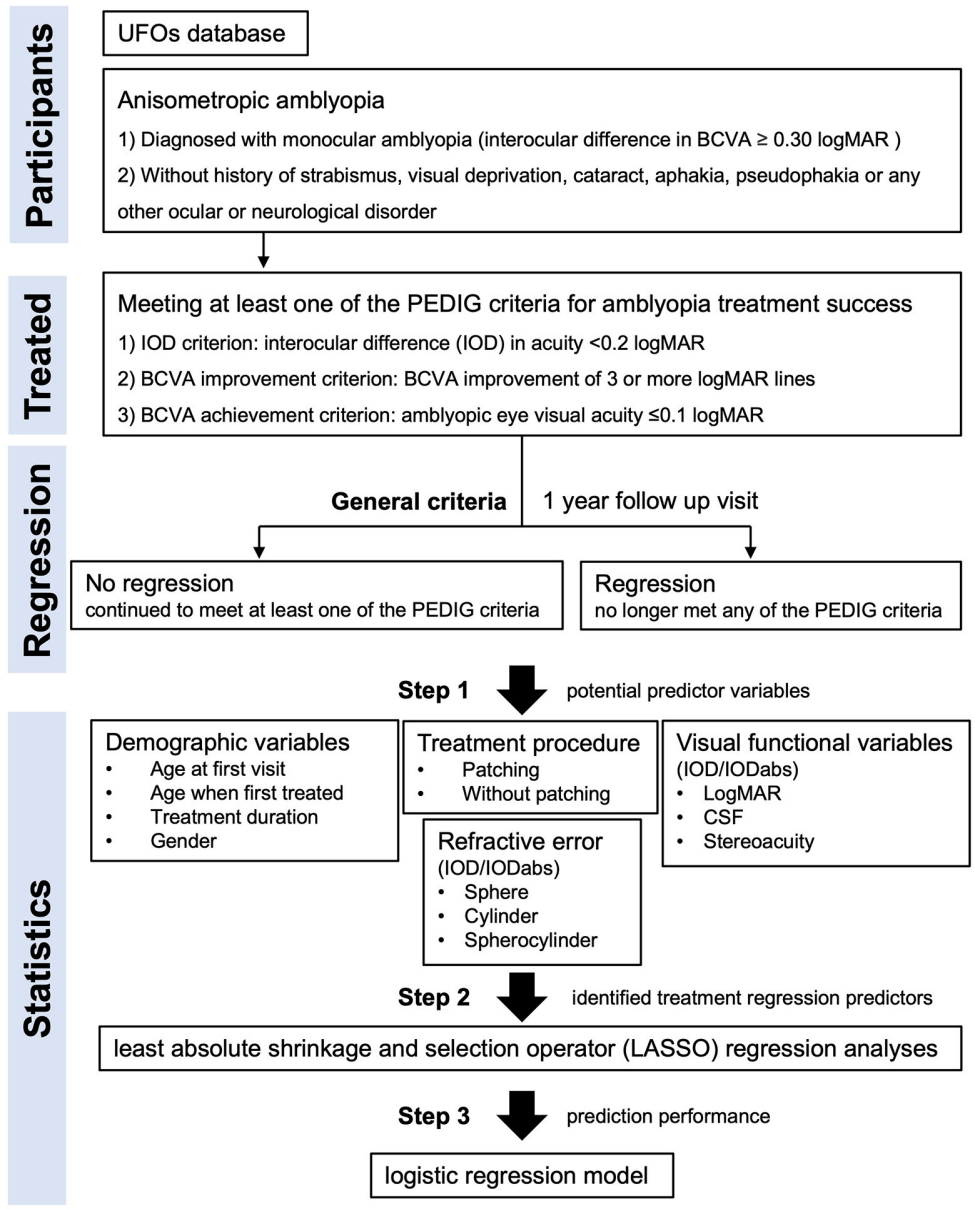
Flowchart depicting the statistical analysis procedure.

The statistical analysis procedure is summarized in Figure 1. The predictor variables represented four different domains. The first domain was patient demographics which included age at first hospital visit (when amblyopia treatment, typically spectacle wear, was started), age when treatment was successful, treatment duration, and sex. The second domain was prior treatment which indicated whether the patient had received both patching and refractive correction or refractive correction alone. The third domain was visual function. Monocular visual acuity measures were included from the first hospital visit and when the patient met the criteria for successful treatment. Improvement from the first hospital visit to the point at which the criteria for successful treatment were met was also calculated for the visual acuity measure. Monocular contrast sensitivities were converted to a single area under the log contrast sensitivity function (AULCSF) value (19). Monocular visual acuity and AULCSF values were entered into the model separately, as an interocular difference (amblyopic eye−fellow eye) and as an absolute interocular difference. Additional visual function measures included stereoacuity (near and far). The fourth domain was refractive error. Variables included spherical, cylindrical refractive error and spherocylinder (the absolute sum of the sphere and cylinder values). Each refractive error variable was entered in the analysis separately, as an interocular difference (amblyopic eye−fellow eye) and an absolute interocular difference.

Eligible participants were split into two groups. The no regression group continued to meet at least one of the PEDIG criteria for 1 year after becoming eligible for the study. The regression group ceased meeting any PEDIG criteria for successful treatment within 1 year of becoming eligible for the study. Predictor variables for treatment regression that differed significantly between the no regression and regression groups were identified using a three-step approach. First, a list of potential predictor variables was created based on available data and variables used in previous studies of treatment regression in amblyopia (Table 1). Second, a least absolute shrinkage and selection operator (LASSO) regression analyses (26) was conducted to identify treatment regression predictors for which a minimal binomial deviance (equal to 0.804, lambda = 0.019) was achieved. This step was necessary because many of the variables were expected to be colinear. Third, predictors that met minimal binomial deviance were included in a logistic regression model to assess sensitivity and specificity for predicting treatment regression. Sensitivity and specificity peak at a value of 1 with perfect sensitivity indicating that no treatment regression cases were labeled as no-regression and perfect specificity indicating that no no-regression cases were labeled as regression cases. For the logistic regression, the data were split into two mutually exclusive datasets, with 70% of records assigned to the training set (*N* = 112) and 30% assigned to the testing set (*N* = 48). As an exploratory analysis, the same statistical procedures were applied separately for each PEDIG criterion. For example, for the interocular difference in acuity <0.2 logMAR criterion (the IOD criterion), only records that met this specific criterion were included in the analysis and regression was defined as no longer meeting this specific criterion.

**Table 1:** Potential predictor variables included in the LASSO regression.

	**Total**	**No regression**	**Regression**	***P*-value**
	***N* = 160**	***N* = 92**	***N* = 68**	
Sex				0.076
Male	87 (54.4)	44 (47.8)	43 (63.2)	
Female	73 (45.6)	48 (52.2)	25 (36.8)	
Previous Patching				0.014*
No	18 (11.2)	5 (5.4)	13 (19.1)	
Yes	142 (88.8)	87 (94.6)	55 (80.9)	
Age at first visit (years)	5.30 (4.50, 6.70)	5.00 (4.30, 5.82)	6.00 (5.00, 8.48)	<0.001*
Age when successfully treated (years)	6.10 (5.40, 7.53)	5.90 (5.27, 6.80)	6.60 (5.57, 9.20)	0.009*
Treatment Duration (years)	0.50 (0.29, 1.15)	0.65 (0.35, 1.29)	0.47 (0.03, 0.76)	0.002*
LogMAR at first visit	0.52 (0.40, 0.80)	0.70 (0.50, 1.00)	0.40 (0.22, 0.52)	<0.001*
LogMAR at first visit-IOD	0.36 (0.22, 0.69)	0.52 (0.34, 0.80)	0.25 (0.13, 0.36)	<0.001*
LogMAR at first visit-IODabs	0.36 (0.26, 0.70)	0.54 (0.35, 0.80)	0.26 (0.16, 0.36)	<0.001*
LogMAR when treated	0.30 (0.20, 0.44)	0.30 (0.20, 0.46)	0.24 (0.20, 0.40)	0.239
LogMAR when treated-IOD	0.20 (0.10, 0.30)	0.20 (0.10, 0.37)	0.18 (0.10, 0.20)	0.185
LogMAR when treated-IODabs	0.20 (0.10, 0.30)	0.20 (0.10, 0.40)	0.18 (0.10, 0.20)	0.100
LogMAR Improvement	0.30 (0.10, 0.40)	0.39 (0.30, 0.50)	0.09 (0.00, 0.25)	<0.001*
AULCSF	0.98 ± 0.32	0.98 ± 0.30	0.97 ± 0.36	0.846
AULCSF-IOD	−0.15 (−0.31, −0.03)	−0.14 (−0.29, −0.03)	−0.16 (−0.40, −0.05)	0.631
AULCSF-IODabs	0.17 (0.07, 0.38)	0.16 (0.06, 0.30)	0.20 (0.10, 0.40)	0.342
Near Random dot (arcsec)	400 (160, 5,000)	400 (160, 5,000)	400 (100, 5,000)	0.339
Distance Randot (arcsec)	5,000 (5,000, 5,000)	5,000 (5000, 5,000)	5,000 (5,000, 5,000)	0.469
Sphere type				0.161
Positive (Hyperopia)	125 (78.1)	76 (82.6)	49 (72.1)	
Negative (Myopia)	35 (21.9)	16 (17.4)	19 (27.9)	
Sphere (D)	4.50 (2.00, 6.00)	4.50 (2.44, 5.81)	4.00 (1.44, 6.00)	0.462
Sphere-IOD (D)	1.50 (0.50, 3.50)	2.00 (0.75, 3.81)	0.88 (0.25, 3.00)	0.023*
Sphere-IODabs (D)	1.88 (0.50, 3.50)	2.00 (0.75, 3.81)	1.25 (0.50, 3.00)	0.017*
Cylinder (D)	1.00 (0.50, 2.00)	1.00 (0.50, 1.75)	1.38 (0.50, 2.31)	0.114
Cylinder-IOD (D)	0.38 (0.00, 1.00)	0.50 (0.00, 1.00)	0.00 (0.00, 0.75)	0.166
Cylinder-IODabs (D)	0.50 (0.19, 1.00)	0.50 (0.25, 1.00)	0.50 (0.00, 1.00)	0.235
Spherocylinder (D)	5.75 (3.44, 7.25)	5.75 (4.00, 7.06)	6.00 (3.00, 7.81)	0.784
Spherocylinder-IOD (D)	2.00 (0.75, 4.06)	2.75 (1.25, 4.50)	1.62 (0.25, 3.06)	0.015*
Spherocylinder-IODabs (D)	2.25 (1.00, 4.06)	2.75 (1.50, 4.50)	1.88 (0.75, 3.06)	0.007*

Data are presented as mean ± SD for normally distributed variables, median (IQR) for non-normally distributed variables, and n (%) for categorical variables. P values were calculated using a t-test, Mann–Whitney U test, or χ2 test. logMAR, logarithm of minimum angle of resolution; AULCSF, the area under the log contrast sensitivity function; Spherocylinder, | sphere | + | cylinder |; IOD, value for the amblyopic eye – value for the fellow eye; IODabs, | IOD |; D, diopter. *Statistically significant difference.

Descriptive statistics were used to summarize the dataset. Categorical variables were expressed as frequencies (percentage), continuous variables as mean ± standard deviation (SD) for normally distributed data, and median (Q1, Q3) for non-normal distributions. Comparisons between the no regression and regression groups were made using *t*-tests or Wilcoxon-Mann-Whitney tests. The chi-square test was used to compare categorical variables. Correlations between included variables were quantified using Spearman's Rho. Differences were considered to be significant at p <0.05. No missing data were reported for age, sex, best corrected visual acuity (BCVA), AULCSF, or refractive error. Missing data for stereoacuity were replaced with median values. All analyses were performed using the R Programming Language (3.6.2).

## Results

**Figure 2:**
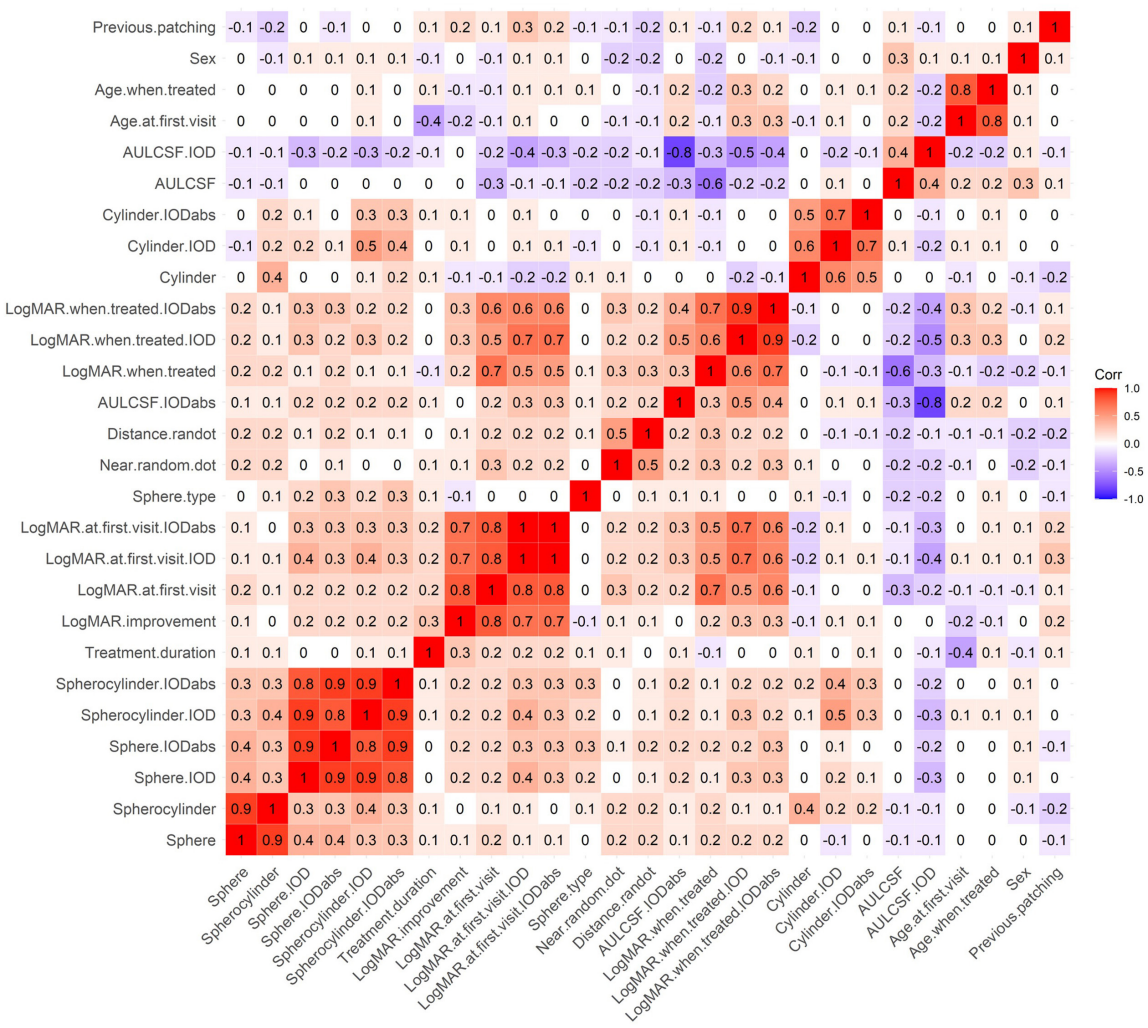
A correlation heatmap of predictor variables. Red represents positive correlations and purple represents negative correlations.

The database search identified 4120 patients with amblyopia of whom 160 met the study inclusion criteria (Table 1). Of the 160 eligible patients, 68 (42.5%) regressed over the course of 1 year. Figure 2 shows a correlation heatmap illustrating the high degree of collinearity between the 27 different predictor variables and the need for two-stage regression analysis.

**Figure 3:**
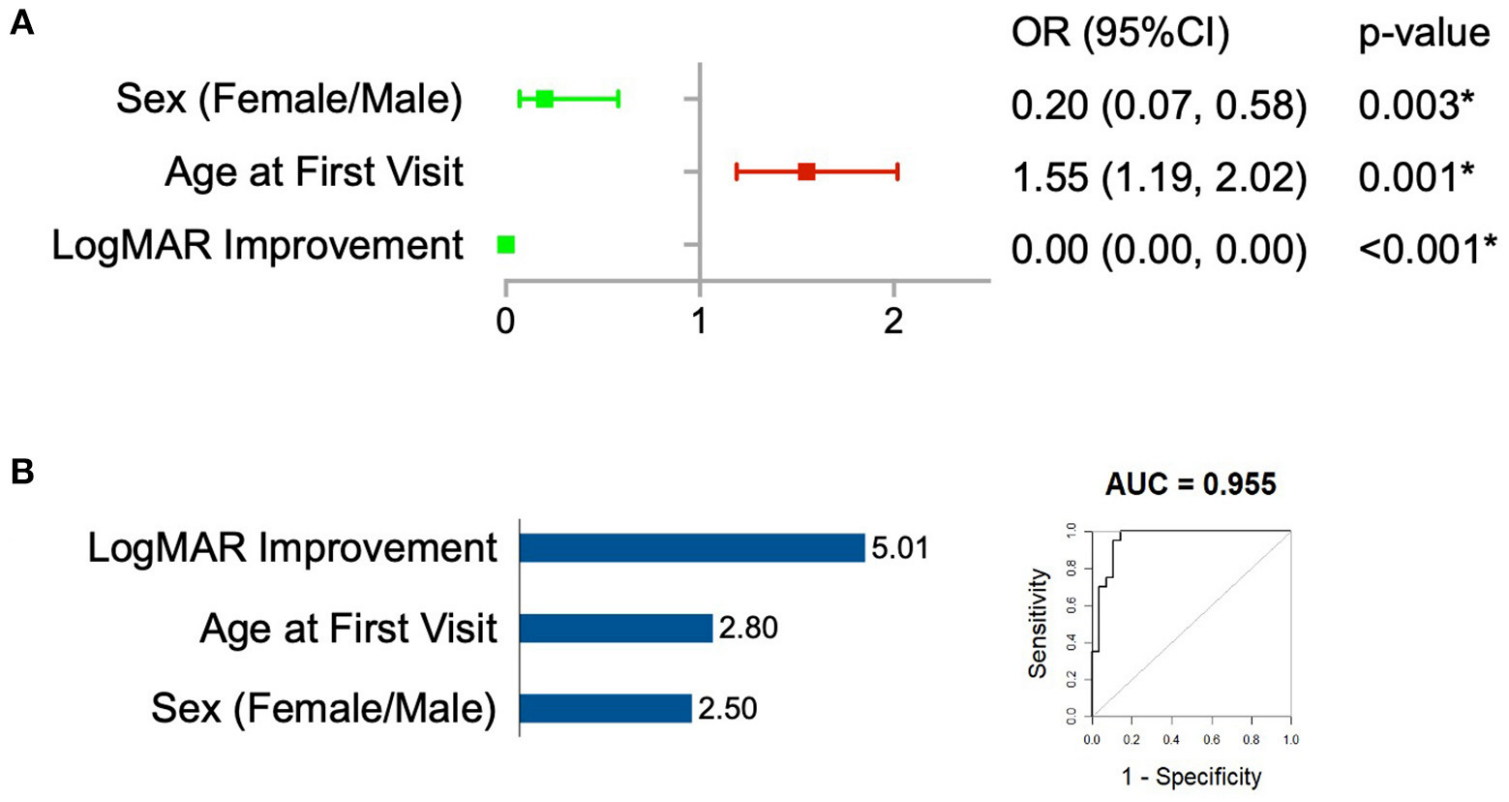
Construction of prognostic models. **(A)** Forest plots of the 3 variables selected by LASSO regression. **(B)** The logistic regression model using variables selected by the LASSO regression (Left, variable importance ranking. Right, The model's ROC curve). The * symbol indicates the statistically significant difference.

LASSO regression (Supplementary Figure S1) identified 3 predictor variables that reached minimal binomial deviance (Figure 3A). These variables indicated that larger improvements in amblyopic eye visual acuity and the initiation of treatment at a younger age were associated with reduced risk of regression. Female sex was also associated with reduced risk of regression. The logistic regression model constructed using these variables (Figure 3B) had good sensitivity (0.75) and specificity (0.93) for predicting amblyopia treatment regression defined as no longer meeting the PEDIG criteria (Table 2).

**Table 2:** Prediction model performance.

**Logistic regression**	**Sensitivity**	**Specificity**	**Kappa**	**Accuracy**	**AUC (95%CI)**
Testing set	0.75	0.93	0.69	0.854	0.955 (0.9–1)

AUC, area under curve; CI, confidence interval.

A breakdown of regression risk as a function of age at first treatment is shown in Table 3. Relative to children who received their first treatment prior to age 6, the odds ratio of regression increased from 2.65 to >8 with increasing age. Notably, the number of children each age bin reduced significantly with increasing age.

**Table 3:** Association between age and the risk of regression.

**Age (years)**	**N**	**Events (%)**	**OR (95%CI)**	***p*-value**
<6	103	33 (32.0)	Reference	
6–8	27	15 (55.6)	2.65 (1.12, 6.29)	0.027
8–10	15	8 (53.3)	2.42 (0.81, 7.25)	0.113
≥10	15	12 (80.0)	8.48 (2.24, 32.12)	0.002
P for Trend				<0.001

To further explore the finding that female sex was associated with reduced risk of regression, the demographic, clinical and treatment data for the regression and no regression groups were summarized by sex (Supplementary Table S1). There were no sex differences in these variables that could explain the association between female sex and lower risk of treatment regression. Of the 160 records included in our study, 130 included an assessment of treatment adherence by the treating physician. There were no differences in the rates of clinician-judged good or poor compliance between boys and girls (4 of 71 boys had poor adherence, vs. 7 of 59 girls, X^2^ = 0.911, *p* = 0.34).

**Figure 4:**
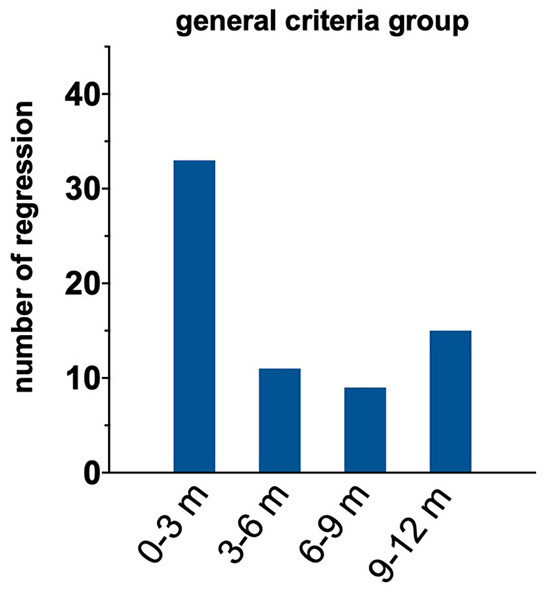
Histogram of time when regression occurs after met treated criteria.

We conducted an additional analysis to identify the time course of treatment regression within our 1-year regression window. Figure 4 shows that most instances of regression occurred within 3 months of successful treatment. There were no differences in the time course of regression between boys and girls (*p* = 0.71). We also examined the magnitude of amblyopic eye visual acuity regression within the treatment regression group. The median magnitude of regression was 1.1 logMAR (IQR 0.04–0.28). The magnitude of regression did not differ as a function of sex (*p* = 0.14).

As described above, the definitions of treatment success and regression used within our primary analysis involved three separate PEDIG criteria: visual acuity IOD <0.2 logMAR, best corrected visual acuity (BCVA) improvement of 3 or more logMAR lines and amblyopic eye visual acuity ≤ 0.1 logMAR. Within the main analysis only one criterion had to be met for treatment to be considered as successful. The treatment regression label was applied when none of the criteria were met by a case that had previously achieved treatment success. Previous studies of treatment regression have used only one criterion to define treatment success and regression. To facilitate comparisons to previous work we conducted separate, pre-planned regression analyses that each used only one of our treatment success / regression criteria (Supplementary material). For example, in the first analysis treatment success was defined as IOD <0.2logMAR and regression as IOD no longer <0.2 logMAR. As expected, the most important predictors varied across these secondary analyses illustrating the importance of using a well-established set of criteria for judging treatment success and regression. However, younger age, greater visual acuity improvement, and less severe spherical anisometropia were each associated with a reduced risk of regression for at least two separate criteria, although these factors did not always reach statistical significance.

## Discussion

In our primary analysis, improvement in amblyopic eye visual acuity had by far the strongest association with risk of regression whereby greater visual acuity improvement was associated with reduced risk of regression. Amblyopic eye visual acuity improved by an average of 0.3 logMAR more in the no-regression group than the regression group. The no regression group also had poorer starting visual acuity and therefore had to exhibit a greater improvement to reach the PEDIG criteria than the regression group. This result is not consistent with previous findings that larger visual acuity improvements were associated with an increased risk of regression (13, 14). One reason for this inconsistency may be that we used a fixed set of clinical thresholds (the PEDIG criteria) to define both successful treatment and regression whereas previous studies have often used relative measures such as defining treatment success as a visual acuity improvement of at least 0.3 logMAR. Therefore, our sample characteristics may have varied from other studies. Another possibility is that we only included patients with anisometropic amblyopia in our sample. This was because a preliminary database search conducted to determine study feasibility indicated that very few patients with other sub-types of amblyopia met the PEDIG criteria for successful treatment. In addition, all participants in the previous studies received patching treatment and treatment was stopped when the treatment success criteria were met. This was not the case for our study where all cases received optical treatment, but only a sub-set received patching. In addition, because this was a retrospective study, treatment was not discontinued when the treatment success criteria were met.

Although it contradicts previous studies (13, 14), our finding that larger visual acuity gains are associated with a reduced risk of regression is intuitive. A larger improvement in amblyopic eye visual acuity indicates a more pronounced change in cortical processing and larger changes in cortical processing may be more robust. In addition, larger gains in visual acuity may encourage subsequent wearing of refractive correction to correct anisometropia which may, in turn, sustain treatment gains.

The association between reduced risk of regression and young age at the initiation of treatment is consistent with previous observations of an inverse relationship between age and treatment efficacy (14, 27). At least two factors could be a play; increased neuroplasticity within younger brains (28, 29) enabling more pronounced and lasting changes in cortical processing and better treatment adherence in young children. Further exploration of this effect revealed a clear increase in the odds ratio of regression with increasing age at first treatment along with a pronounced reduction in the number of children in the older age groups. This change in the number of children in each age group may reflect a smaller proportion of older children reaching the criteria for treatment success, possibly because of reduced neuroplasticity and / or treatment adherence.

Female sex was associated with a reduced risk of regression. Exploratory analyses did not identify any demographic or clinical factors that could satisfactorily explain this effect. In addition, the time course and magnitude of regression was similar between females and males. If later studies replicate this finding, there are several possible explanations. Sex differences in childhood brain development are well established and involve differences in the rate and magnitude of brain structure changes (30–32). Amblyopia treatment, which relies critically on neuroplasticity within the brain, may interact with these developmental changes leading to sex-differences in the risk of treatment regression. Alternatively, environmental differences between boys and girls including engagement in activities with varying visual demands may also influence treatment regression. Sex differences in visual activities are affected by many factors and in this regard our data only relate to children in southern China. In general, little is known about sex differences in human visual development and critical period visual cortex neuroplasticity (33). Our result suggests that this knowledge gap requires attention.

Our work builds upon previous studies of treatment regression in several ways: (1) we had access to 27 different potential predictors of treatment regression that included unique measures such as psychophysical assessments of contrast sensitivity, (2) we adopted an accepted set of clinical criteria for identifying treatment success and regression, and (3) our statistical approach allowed us to include each predictor in the modeling process and identify those that explained the most variance in treatment regression. This comprehensive approach provided a robust identification of factors that predict treatment regression in anisometropic amblyopia.

## Conclusion

Multiple factors are associated with risk of regression following successful treatment for anisometropic amblyopia. However, larger visual acuity gains and a younger age of treatment have the strongest association with reduced regression risk. These findings highlight the importance of vision screening for early detection and treatment of amblyopia. In addition, our results suggest that male patients may require additional monitoring to protect against a heightened risk of treatment regression.

## Data availability statement

The raw data supporting the conclusions of this article will be made available by the authors, without undue reservation.

## Ethics statement

The studies involving human participants were reviewed and approved by Institutional Ethics Committee of Zhongshan Ophthalmic Center, Sun Yat-Sen University. Written informed consent to participate in this study was provided by the participants' legal guardian/next of kin.

## Author contributions

JLi and BT contributed to the project design, data analysis, and the revision of the manuscript. YJ, JLiu, and QY contributed to the project design, data analysis, and drafted the manuscript. SZ, LF, ZX, YZhu, YH, YZho, XC, YY, and RJ contributed to data collection and manuscript discussion. All authors contributed to the article, read, and approved the submitted version.

## Funding

JL was supported by National Key Research & Development Project (2020YFC2003905). BT was supported by the Hong Kong Special Administrative Region Government and InnoHK, CIHR grant 156174 and NSERC RPIN-05394. The sponsor or funding organization had no role in the design or conduct of this research.

## Conflict of interest

The authors declare that the research was conducted in the absence of any commercial or financial relationships that could be construed as a potential conflict of interest.

## Publisher's note

All claims expressed in this article are solely those of the authors and do not necessarily represent those of their affiliated organizations, or those of the publisher, the editors and the reviewers. Any product that may be evaluated in this article, or claim that may be made by its manufacturer, is not guaranteed or endorsed by the publisher.
