# Supplementary material for: Factors predicting regression of visual acuity following successful treatment of anisometropic amblyopia
Source: Front Med (Lausanne). 2022 Oct 31;9:1013136. doi: 10.3389/fmed.2022.1013136 (PMC9659723; doi:10.3389/fmed.2022.1013136)
Supplement: Supplementary file 1 [file Data_Sheet_1.PDF]

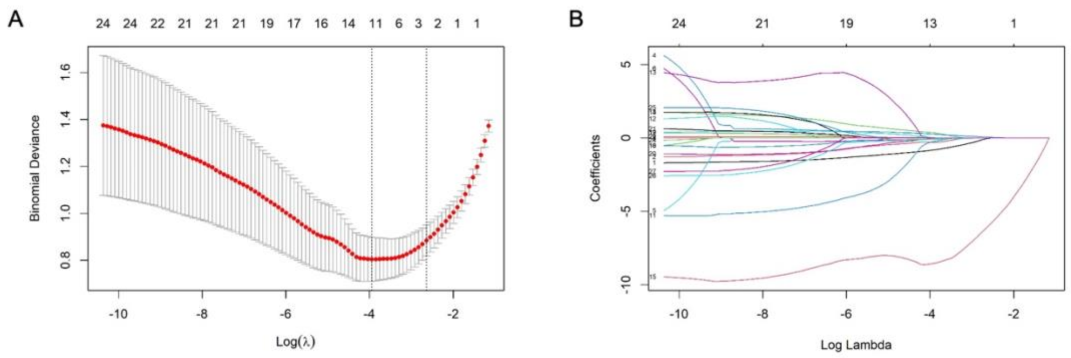

Supplementary Figure 1. The LASSO prognostic model in the general criteria group. (A) Binomial deviance of the LASSO model with different log lambdas. (B) LASSO coefficient profiles of the prognostic factors predicting amblyopia regression.

Supplementary Table 1. Sex subgroup comparison with regression and no regression groups separated.

Variables	Male Regression Group (n=87)		Female Regression Group (n=73)		Multiple groups	Male No vs Yes	Female No vs Yes	Yes vs Yes	No vs No
	Yes (n=43)	No (n=44)	Yes (n=25)	No (n=48)					
**Age at first visit (years)**	5.80 (4.80, 8.20)	4.80 (4.33, 5.55)	7.10 (5.30, 10.30)	5.30 (4.30, 6.80)	H=21.197 P<0.001*	Z=-3.105 P=0.002*	Z=-2.844 P=0.004*	Z=-1.731 P=0.083	Z=-1.611 P=0.107
**Age when successfully treated (years)**	6.50 (5.50, 8.80)	5.60 (5.10, 6.45)	7.10 (5.60, 10.65)	6.25 (5.33, 8.05)	H=11.752 P=0.008*	Z=-2.715 P=0.007*	Z=-1.506 P=0.132	Z=-0.948 P=0.343	Z=-2.066 P=0.039*
**Treatment Duration (years)**	0.50 (0.29, 1.13)	0.69 (0.33, 1.34)	0.31 (0.16, 0.56)	0.57 (0.33, 1.29)	H=17.987 P<0.001*	Z=-1.439 P=0.150	Z=-3.709 P<0.001*	Z=-2.834 P=0.005*	Z=-0.582 P=0.560
**Sphere Type**									
**Hyperopia**	31 (72.1)	37 (84.1)	18 (72.0)	39 (81.3)	X^2=2.655 P=0.448	/			
**Myopia**	12 (27.9)	7 (15.9)	7 (28.0)	9 (18.8)					
**Sphere (absolute), D**	4.50 (2.00, 6.00)	4.63 (2.25, 5.75)	3.25 (0.38, 5.75)	4.38 (2.56, 6.00)	H=2.295 P=0.513	/			
**Cylinder (absolute), D**	1.50 (0.75, 2.50)	1.00 (0.56, 1.69)	0.75 (0.38, 2.13)	1.00 (0.50, 1.75)	H=6.886 P=0.076	/			
**Sphere (Hyperopia), D n=125**	n=31 5.00 (1.75, 6.50)	n=37 4.75 (2.38, 5.75)	n=18 3.50 (0.44, 5.38)	n=39 3.75 (2.00, 5.25)	H=3.739 P=0.291	/			
**Sphere (Myopia), D n=35**	n=12 3.13 (2.06, 5.44)	n=7 4.50 (0.75, 5.75)	n=7 2.75 (0.25, 12.50)	n=9 6.50 (6.00, 8.63)	H=10.454 P=0.015*	Z=-0.170 P=0.902	Z=-1.431 P=0.174	Z=-0.466 P=0.650	Z=-2.546 P=0.008*

NOTE. Data are presented as median (IQR) for non-normally distributed variables and n (%) for categorical variables. P values were calculated using a Kruskal-Wailis H test or χ2 test for comparison among 4 groups, and Mann–Whitney U test for comparison between two groups. D = diopter. * Statistically significant difference.

Exploratory analyses conducted separately for each regression criterion.

Prognostic model for the interocular visual acuity difference (IOD) criterion group.

Supplementary Table 2. Potential predictor variables included in the LASSO regression in the IOD criterion group.

	Total	No regression	Regression	P-value
	n = 170	n = 78	n = 92	
Sex				0.290
Male	100 (58.8)	42 (53.8)	58 (63.0)	
Female	70 (41.2)	36 (46.2)	34 (37.0)	
Previous Patching				0.015*
No	24 (14.1)	5 (6.4)	19 (20.7)	
Yes	146 (85.9)	73 (93.6)	73 (79.3)	
Age at first visit (years)	5.20 (4.50, 6.18)	4.85 (4.23, 5.70)	5.60 (4.90, 6.70)	0.003*
Age when criterion was met (years)	6.10 (5.40, 7.20)	5.90 (5.32, 6.80)	6.25 (5.40, 7.55)	0.138
Treatment Duration (years)	0.60 (0.31, 1.18)	0.82 (0.36, 1.31)	0.48 (0.29, 1.08)	0.014*
LogMAR at first visit	0.46 (0.30, 0.68)	0.50 (0.40, 0.70)	0.40 (0.23, 0.56)	0.002*
LogMAR at first visit-IOD	0.30 (0.10, 0.44)	0.36 (0.30, 0.50)	0.20 (0.02, 0.35)	<0.001*
LogMAR at first visit-IODabs	0.34 (0.22, 0.48)	0.38 (0.30, 0.50)	0.30 (0.16, 0.46)	<0.001*
LogMAR when treated	0.22 (0.18, 0.30)	0.20 (0.11, 0.30)	0.26 (0.20, 0.34)	0.001*
LogMAR when treated-IOD	0.12 (0.02, 0.20)	0.10 (0.04, 0.20)	0.16 (0.00, 0.20)	0.424
LogMAR when treated-IODabs	0.16 (0.10, 0.20)	0.10 (0.08, 0.20)	0.18 (0.10, 0.20)	0.001*
LogMAR Improvement	0.20 (0.06, 0.35)	0.28 (0.18, 0.42)	0.10 (0.00, 0.24)	<0.001*
AULCSF	1.05 ± 0.30	1.09 ± 0.27	1.03 ± 0.31	0.193
AULCSF-IOD	-0.08 (-0.21, 0.05)	-0.06 (-0.17, 0.04)	-0.10 (-0.29, 0.07)	0.404
AULCSF-IODabs	0.15 (0.06, 0.25)	0.11 (0.05, 0.19)	0.19 (0.09, 0.38)	<0.001*
Near Random dot (arcsec)	200 (100, 5000)	200 (100, 5000)	200 (100, 5000)	0.201
Distance Randot (arcsec)	5000 (5000, 5000)	5000 (1550, 5000)	5000 (5000, 5000)	0.013*
Sphere Type				0.121
Positive (Hyperopia)	129 (75.9)	64 (82.1)	65 (70.7)	
Negative (Myopia)	41 (24.1)	14 (17.9)	27 (29.3)	
Sphere (D)	4.38 (2.00, 5.75)	4.00 (2.06, 5.25)	4.62 (2.00, 6.00)	0.248
Sphere-IOD (D)	1.25 (0.00, 2.50)	1.25 (0.25, 2.88)	0.75 (-0.25, 2.50)	0.153
Sphere-IODabs (D)	1.75 (0.50, 3.00)	1.50 (0.75, 3.00)	1.75 (0.50, 2.81)	0.588
Cylinder (D)	1.25 (0.56, 2.00)	1.00 (0.50, 1.75)	1.50 (0.75, 2.25)	0.010*
Cylinder-IOD (D)	0.25 (0.00, 0.75)	0.50 (0.00, 0.94)	0.00 (0.00, 0.75)	0.022*
Cylinder-IODabs (D)	0.50 (0.00, 1.00)	0.50 (0.25, 1.00)	0.25 (0.00, 0.81)	0.032*
Spherocylinder (D)	5.75 (3.50, 7.25)	5.00 (3.56, 6.50)	6.12 (3.44, 8.00)	0.031*

**Spherocylinder-IOD (D)**	1.77 ± 2.58	2.17 ± 2.24	1.43 ± 2.80	0.061
**Spherocylinder-IODabs (D)**	2.12 (1.25, 3.50)	2.25 (1.31, 3.69)	2.00 (1.19, 3.06)	0.475

NOTE. The IOD criterion group stands for the criterion that interocular difference in acuity <0.23 logMAR. Data are presented as mean ± SD for normally distributed variables, median (IQR) for non-normally distributed variables, and n (%) for categorical variables. P values were calculated using a t test, Mann–Whitney U test, or χ2 test.

Abbreviations: logMAR = logarithm of minimum angle of resolution; AULCSF = the area under the log contrast sensitivity function; Spherocylinder = | sphere | + | cylinder |; IOD= value for the amblyopic eye – value for the fellow eye; IODabs = | IOD |; D = diopter. * Statistically significant difference.

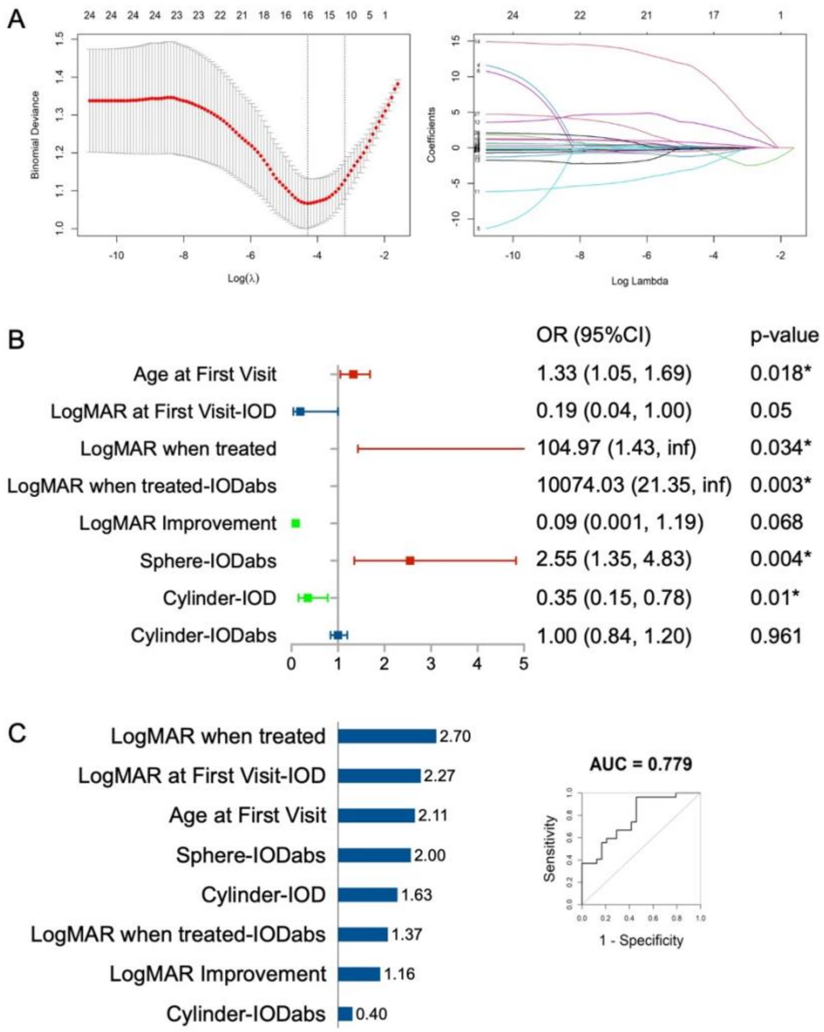

Supplementary Figure 2. Construction of prognostic model in the IOD criterion group. (A) Left, Binomial deviance of the LASSO model with different log (lambda)s. Right, LASSO coefficient profiles of the prognostic factors predicting amblyopia regression. (B) The forest plots of the 8 variables screened out by LASSO regression. (C) The logistic regression model for amblyopia regression using variables selected by LASSO (Left, variable importance ranking. Right, AUC of the model.).

Supplementary Table 3. Prediction model performance in the IOD criterion group.

Logistic Regression	Sensitivity	Specificity	Kappa	Accuracy	AUC (95%CI)
**Testing Set**	0.59	0.79	0.38	0.69	0.779 (0.653-0.906)

NOTE. AUC = area under curve, CI = confidence interval.

Prognostic model for the best corrected visual acuity (BCVA) improvement criterion group.

Supplementary Table 4. Potential predictor variables included in the LASSO regression in the BCVA improvement criterion group.

	Total	No regression	Regression	P-value
	**n = 122**	**n = 94**	**n = 28**	
**Sex**				0.074
**Male**	67 (54.9)	47 (50.0)	20 (71.4)	
**Female**	55 (45.1)	47 (50.0)	8 (28.6)	
**Previous Patching**				0.012*
**No**	10 (8.2)	4 (4.3)	6 (21.4)	
**Yes**	112 (91.8)	90 (95.7)	22 (78.6)	
**Age at first visit (years)**	5.20 (4.40, 6.10)	5.05 (4.32, 5.97)	5.30 (4.88, 7.08)	0.11
**Age when criterion was met (years)**	6.15 (5.40, 7.20)	5.95 (5.30, 7.00)	6.95 (5.77, 8.43)	0.041*
**Treatment Duration (years)**	0.82 (0.44, 1.34)	0.83 (0.42, 1.31)	0.80 (0.46, 1.46)	0.686
**LogMAR at first visit**	0.78 (0.52, 1.00)	0.80 (0.70, 1.20)	0.50 (0.40, 0.80)	<0.001*
**LogMAR at first visit-IOD**	0.61 (0.36, 0.80)	0.65 (0.44, 0.85)	0.38 (0.24, 0.57)	0.003*
**LogMAR at first visit-IODabs**	0.62 (0.40, 0.81)	0.66 (0.47, 0.85)	0.49 (0.30, 0.73)	0.017*
**LogMAR when treated**	0.30 (0.15, 0.50)	0.32 (0.18, 0.50)	0.20 (0.10, 0.50)	0.126
**LogMAR when treated-IOD**	0.20 (0.10, 0.40)	0.20 (0.10, 0.40)	0.18 (0.00, 0.37)	0.302
**LogMAR when treated-IODabs**	0.24 (0.12, 0.44)	0.25 (0.10, 0.42)	0.21 (0.16, 0.54)	0.604
**LogMAR Improvement**	0.40 (0.32, 0.52)	0.42 (0.36, 0.58)	0.30 (0.30, 0.34)	<0.001*
**AULCSF**	1.01 ± 0.33	1.02 ± 0.31	0.96 ± 0.40	0.368
**AULCSF-IOD**	-0.16 (-0.48, 0.01)	-0.16 (-0.47, -0.00)	-0.17 (-0.48, 0.14)	0.650
**AULCSF-IODabs**	0.22 (0.09, 0.50)	0.19 (0.07, 0.49)	0.26 (0.16, 0.54)	0.193
**Near Random dot (arcsec)**	400 (160, 5000)	400 (160, 5000)	400 (145, 5000)	0.497
**Distance Randot (arcsec)**	5000 (5000, 5000)	5000 (5000, 5000)	5000 (5000, 5000)	0.868
**Sphere Type**				0.585
**Positive (Hyperopia)**	98 (80.3)	74 (78.7)	24 (85.7)	
**Negative (Myopia)**	24 (19.7)	20 (21.3)	4 (14.3)	
**Sphere (D)**	4.50 (2.25, 6.00)	4.50 (2.50, 6.00)	3.75 (1.75, 6.00)	0.446

**Sphere-IOD (D)**	2.00 (0.75, 3.75)	2.00 (0.75, 3.94)	0.88 (-0.06, 3.12)	0.052
**Sphere-IODabs (D)**	2.00 (0.75, 3.75)	2.00 (1.00, 3.94)	1.38 (0.50, 3.12)	0.075
**Cylinder (D)**	1.00 (0.56, 2.00)	1.00 (0.50, 1.75)	1.38 (1.00, 2.00)	0.056
**Cylinder-IOD (D)**	0.50 (0.00, 1.00)	0.50 (0.00, 1.00)	0.38 (-0.06, 1.00)	0.681
**Cylinder-IODabs (D)**	0.50 (0.25, 1.00)	0.50 (0.25, 1.00)	0.62 (0.25, 1.25)	0.558
**Spherocylinder (D)**	5.57 ± 2.55	5.58 ± 2.50	5.55 ± 2.76	0.962
**Spherocylinder-IOD (D)**	2.50 (1.00, 4.25)	2.75 (1.31, 4.50)	1.75 (0.25, 3.75)	0.097
**Spherocylinder-IODabs (D)**	2.62 (1.31, 4.25)	2.75 (1.50, 4.50)	2.00 (0.50, 3.75)	0.097

NOTE. The BCVA improvement criterion group stands for the criterion that BCVA improvement of 3 or more logMAR lines in the affected eyes. Data are presented as mean ± SD for normally distributed variables, median (IQR) for non-normally distributed variables, and n (%) for categorical variables. P values were calculated using a t test, Mann–Whitney U test, or χ2 test. Abbreviations: logMAR = logarithm of minimum angle of resolution; AULCSF = the area under the log contrast sensitivity function; Spherocylinder = | sphere | + | cylinder |; IOD= value for the amblyopic eye – value for the fellow eye; IODabs = | IOD |; D = diopter. * Statistically significant difference.

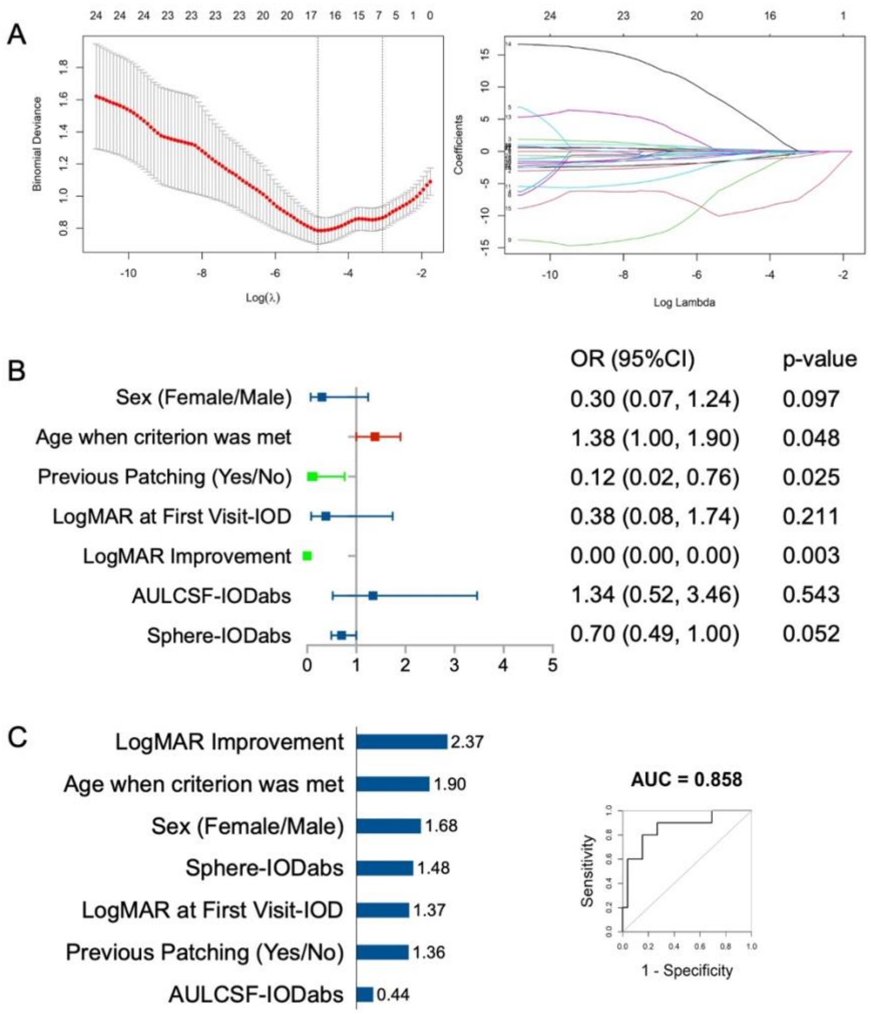

Supplementary Figure 3. Construction of prognostic model in the BCVA improvement criterion group. (A) Left, Binomial deviance of the LASSO model with different log (lambda)s. Right, LASSO coefficient profiles of the prognostic factors predicting amblyopia regression. (B) The forest plots of the 7 variables

screened out by LASSO regression. (C) The logistic regression model for amblyopia regression using variables selected by LASSO (Left, variable importance ranking. Right, AUC of the model.).

Supplementary Table 5. Prediction model performance in the BCVA improvement criterion group.

Logistic Regression	Sensitivity	Specificity	Kappa	Accuracy	AUC (95%CI)
**Testing Set**	0.500	0.962	0.536	0.833	0.858 (0.709-1)

NOTE. AUC = area under curve, CI = confidence interval.

Prognostic model for the BCVA achievement criterion group.
Supplementary Table 6. Potential predictor variables included in the LASSO regression in the BCVA achievement criterion group.

	Total	No regression	Regression	P-value
	n = 47	n = 20	n = 27	
**Sex**				0.796
**Male**	21 (44.7)	8 (40.0)	13 (48.1)	
**Female**	26 (55.3)	12 (60.0)	14 (51.9)	
**Previous Patching**				0.870
**No**	17 (36.2)	8 (40.0)	9 (33.3)	
**Yes**	30 (63.8)	12 (60.0)	18 (66.7)	
**Age at first visit (years)**	5.30 (4.55, 6.75)	4.80 (4.07, 6.15)	5.40 (4.85, 7.20)	0.102
**Age when criterion was met (years)**	6.50 (5.60, 7.80)	6.60 (5.80, 7.05)	6.30 (5.60, 8.40)	0.613
**Treatment Duration (years)**	1.10 (0.40, 1.55)	1.10 (0.48, 2.02)	0.90 (0.20, 1.40)	0.181
**LogMAR at first visit**	0.38 (0.18, 0.50)	0.40 (0.32, 0.52)	0.20 (0.13, 0.45)	0.008*
**LogMAR at first visit-IOD**	0.20 (-0.01, 0.32)	0.30 (-0.33, 0.33)	0.16 (0.01, 0.30)	0.470
**LogMAR at first visit-IODabs**	0.30 (0.17, 0.41)	0.33 (0.30, 0.47)	0.20 (0.05, 0.34)	0.001*
**LogMAR when treated**	0.10 (0.06, 0.10)	0.10 (0.06, 0.10)	0.10 (0.06, 0.10)	0.254
**LogMAR when treated-IOD**	0.06 (-0.04, 0.10)	0.03 (-0.10, 0.10)	0.08 (-0.03, 0.10)	0.419
**LogMAR when treated-IODabs**	0.10 (0.06, 0.10)	0.10 (0.05, 0.12)	0.10 (0.06, 0.10)	0.566
**LogMAR Improvement**	0.28 (0.11, 0.41)	0.34 (0.26, 0.42)	0.18 (0.03, 0.40)	0.013*
**AULCSF**	1.29 ± 0.29	1.27 ± 0.29	1.30 ± 0.29	0.770
**AULCSF-IOD**	0.04 ± 0.27	0.08 ± 0.28	0.01 ± 0.26	0.380
**AULCSF-IODabs**	0.15 (0.06, 0.24)	0.10 (0.05, 0.24)	0.16 (0.11, 0.26)	0.196
**Near Random dot (arcsec)**	160 (63, 2700)	160 (63, 5000)	160 (63, 300)	0.710
**Distance Randot (arcsec)**	5000 (400, 5000)	5000 (350, 5000)	5000 (400, 5000)	0.968
**Sphere Type**				0.070

**Positive (Hyperopia)**	41 (87.2)	20 (100.0)	21 (77.8)	
**Negative (Myopia)**	6 (12.8)	0 (0.0)	6 (22.2)	
**Sphere (D)**	3.36 ± 2.26	3.54 ± 2.08	3.22 ± 2.41	0.641
**Sphere-IOD (D)**	0.25 (0.00, 1.25)	0.75 (-0.31, 1.31)	0.25 (0.00, 0.88)	0.738
**Sphere-IODabs (D)**	0.75 (0.25, 1.62)	1.12 (0.69, 1.75)	0.50 (0.12, 1.50)	0.093
**Cylinder (D)**	1.00 (0.50, 1.88)	1.00 (0.50, 1.31)	1.00 (0.00, 2.00)	0.913
**Cylinder-IOD (D)**	0.25 (0.00, 0.75)	0.50 (-0.06, 0.75)	0.25 (0.00, 0.75)	0.283
**Cylinder-IODabs (D)**	0.50 (0.25, 0.75)	0.62 (0.50, 0.88)	0.50 (0.12, 0.75)	0.201
**Spherocylinder (D)**	4.48 ± 2.33	4.65 ± 2.05	4.36 ± 2.55	0.680
**Spherocylinder-IOD (D)**	1.00 ± 1.98	1.16 ± 2.14	0.88 ± 1.89	0.634
**Spherocylinder-IODabs (D)**	1.25 (0.88, 2.38)	1.62 (1.19, 2.50)	1.00 (0.50, 2.00)	0.092

NOTE. The BCVA achievement criterion group stands for the criterion that amblyopic eye visual acuity ≤0.18 logMAR. Data are presented as mean ± SD for normally distributed variables, median (IQR) for non-normally distributed variables, and n (%) for categorical variables. P values were calculated using a t test, Mann–Whitney U test, or χ2 test. Abbreviations: logMAR = logarithm of minimum angle of resolution; AULCSF = the area under the log contrast sensitivity function; Spherocylinder = | sphere | + | cylinder |; IOD= value for the amblyopic eye – value for the fellow eye; IODabs = | IOD |; D = diopter. * Statistically significant difference.

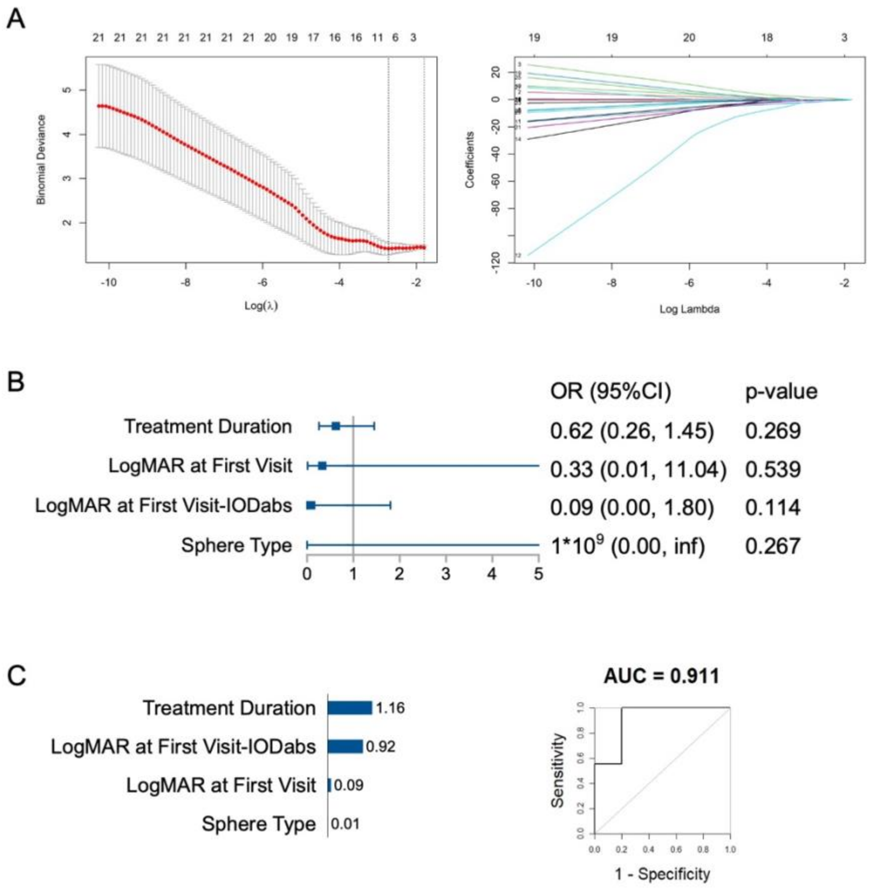

Supplementary Figure 4. Construction of prognostic model in the BCVA achievement criterion group. (A) Left, Binomial deviance of the LASSO model with different log (lambda)s. Right, LASSO coefficient profiles of the prognostic factors predicting amblyopia regression. (B) The forest plots of the 8 variables

screened out by LASSO regression. (C) The logistic regression model for amblyopia regression using variables selected by LASSO (Left, variable importance ranking. Right, AUC of the model.).

Supplementary Table 7. Prediction model performance in the BCVA achievement criterion group.

Logistic Regression	Sensitivity	Specificity	Kappa	Accuracy	AUC (95%CI)
**Testing Set**	0.89	0.80	0.69	0.857	0.911 (0.724-1)

NOTE. AUC = area under curve, CI = confidence interval.

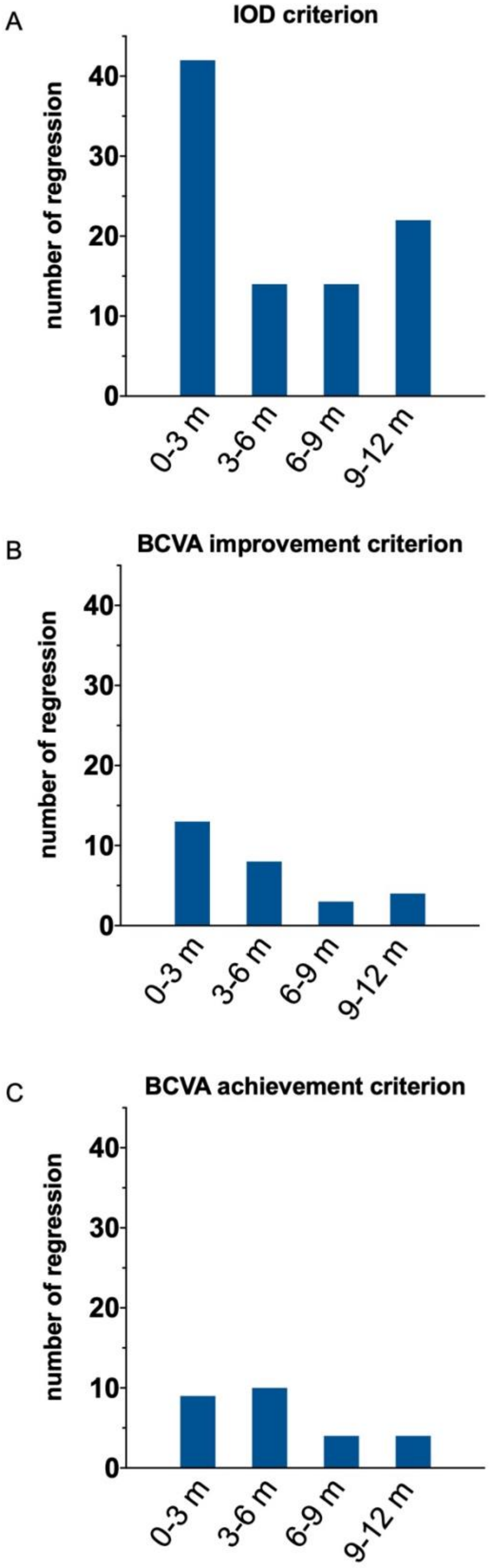

Supplementary Figure 5. Histogram of time when regression occurred for each separate treatment criterion.
